# Correction: Influence of time dependent laser-irradiation for tuning the linear–nonlinear optical response of quaternary Ag_10_In_15_S_15_Se_60_ films for optoelectronic applications

**DOI:** 10.1039/d3ra90094k

**Published:** 2023-09-26

**Authors:** Abinash Parida, D. Alagarasan, R. Ganesan, Sagar Bisoyi, R. Naik

**Affiliations:** a Department of Engineering and Materials Physics, ICT-IOC Bhubaneswar 751013 India ramakanta.naik@gmail.com; b Department of Physics, Indian Institute of Science Bengaluru 560012 India; c Department of Physics, NITTE Meenakshi Institute of Technology Yelahanka Bengaluru 560064 India; d Department of Physics, School of Applied Science, KIIT Deemed to be University Bhubaneswar 751024 India

## Abstract

Correction for ‘Influence of time dependent laser-irradiation for tuning the linear–nonlinear optical response of quaternary Ag_10_In_15_S_15_Se_60_ films for optoelectronic applications’ by Abinash Parida *et al.*, *RSC Adv.*, 2023, **13**, 4236–4248, https://doi.org/10.1039/D2RA07981J.

The authors regret that the formula used for electrical conductivity was not dimensionally correct in Section 3.4.3 of the original article. Thus, the corresponding Fig. 6(c) was also incorrect for this article.

Consequently, Section 3.4.3 and Fig. 6 have been adjusted, and this is detailed below. An independent expert has viewed the corrected data and image and has concluded that they are consistent with the discussions and conclusions presented.

**3.4.3 Optical density, optical, and electrical conductivity**

The optical density (OD) of the materials is a measure of how well they can absorb electromagnetic radiation. The formula OD = *α* × *t* is used to get the OD value. According to Fig. 6(a), the optical density varies with wavelength and decreases over the course of irradiation time. The decreasing behaviour of the OD is because of the reduction in the *α* value. The optical and electrical conductivity of the material can provide detailed knowledge about the electronic states, and the optical conductivity could be determined by using the values of *α*, *n*, and *λ* in the relation, 
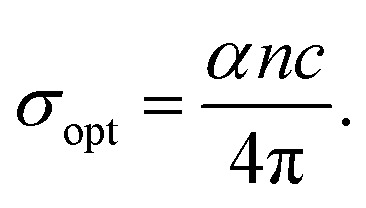


Gomma *et al.* have recently reported the formula for the calculation of electrical conductivity as follows,^1^
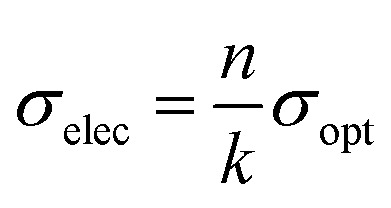
where ‘*n*’ is the refractive index and ‘*k*’ is the extinction coefficient. The conductance of charge carriers because of optical excitation is represented by the *σ*_opt_. Fig. 6(b) and (c) show the fluctuation of these two conductivities (*σ*_opt_ and *σ*_ele_) at various irradiation times. Both the conductivities decreased with the irradiation time, that is because of loss in ‘*α*’ and density of localized defect states caused by the irradiation.

**Fig. 6:**
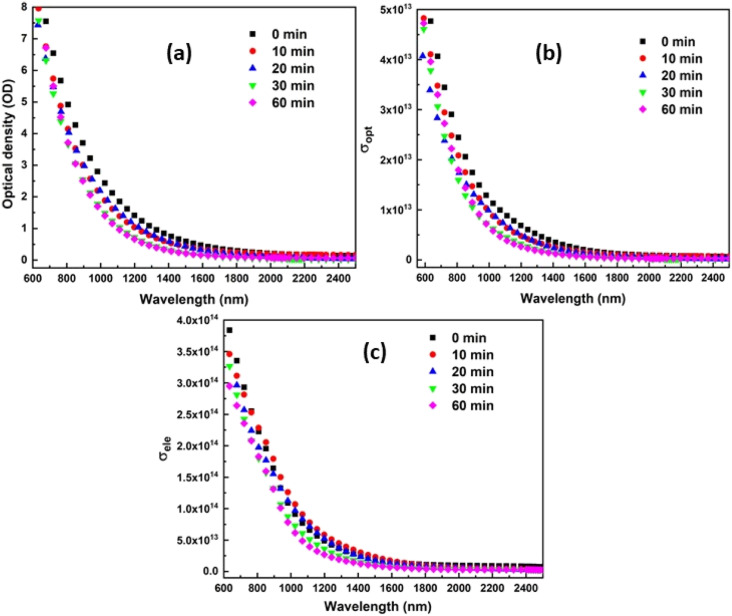
Variation of (a) OD with wavelength, (b) optical conductivity and (c) electrical conductivity with the wavelength of laser irradiated Ag_10_In_15_S_15_Se_60_ films with different time duration.

The Royal Society of Chemistry apologises for these errors and any consequent inconvenience to authors and readers.

## Supplementary Material
